# Q69 (an *E. faecalis*-Infecting Bacteriophage) As a Biocontrol Agent for Reducing Tyramine in Dairy Products

**DOI:** 10.3389/fmicb.2016.00445

**Published:** 2016-04-05

**Authors:** Victor Ladero, Carolina Gómez-Sordo, Esther Sánchez-Llana, Beatriz del Rio, Begoña Redruello, María Fernández, M. Cruz Martín, Miguel A. Alvarez

**Affiliations:** Department of Technology and Biotechnology of Dairy Products, Dairy Research Institute, Instituto de Productos Lácteos de Asturias – Consejo Superior de Investigaciones Científicas (IPLA–CSIC)Villaviciosa, Spain

**Keywords:** biogenic amines, tyramine, characterization, cheese, *Enterococcus faecalis*, bacteriophage Q69

## Abstract

Biogenic amines (BAs) are low molecular weight nitrogenous compounds with biological activity, formed from amino acids by decarboxylation. BAs are naturally present in all living organisms playing essential roles. However, their accumulation in food through the metabolic activity of certain microorganisms constitutes a toxicological hazard. Among foods, cheeses accumulate some of the highest concentrations of BAs since they provide an ideal environment for their accumulation. Most of the methods proposed for reducing BAs in cheese, such as milk pasteurization, have not only failed to completely solve the problem, they also affect non-BA producing lactic acid bacteria, i.e., the bacteria that participate in the development of the organoleptic characteristics of cheese. Novel technologies specifically targeted against BA producers are therefore needed to control BA accumulation. Bacteriophages have been proposed as agents for specifically controlling the presence of foodborne pathogens in food. Due to its specificity, they could be used as a biotechnological tool targeted to reduce the population of BA-producing bacteria. The present work reports the isolation, from cheese, and the characterization of bacteriophage Q69, which infects specifically *Enterococcus faecalis*, the species mainly responsible of the accumulation of the BA tyramine in foods. Furthermore, its capacity to reduce the accumulation of tyramine in different conditions –including a model cheese- was proven. The obtained results open up the possibility of use bacteriophages to prevent BA accumulation in fermented foods.

## Introduction

Biogenic amines, low molecular weight (MW) nitrogenous compounds with biological activity, are formed from amino acids by decarboxylation. They are present in all living organisms, in which they play essential physiological roles. However, the metabolic activity of certain microorganisms can cause them to accumulate in foods, constituting a toxicological hazard (EFSA, 2011). The ingestion of such foods can lead to headache, hypertension and rashes, etc (Ladero et al., 2010a).

Foods likely to have high BA concentrations include fish, fish products, and fermented foods and beverages, including dairy products, fermented meat and vegetables, wine, cider and beer (ten Brink et al., 1990; Stratton et al., 1991; Spano et al., 2010). Cheese has some of the highest BA concentrations (EFSA, 2011) because it constitutes an ideal environment for their production and accumulation (Linares et al., 2012). The milk used in cheesemaking is generally not sterile and contain BA-producing bacteria that belong to the normal cheese microbiota. The physico-chemical conditions (pH, temperature, etc.) for optimal cheese manufacturing favor the production of BAs. In addition, large amounts of amino acids – the substrates for BA production – are released over ripening (Linares et al., 2012).

The bacteria responsible for BA accumulation vary with food type. The BA producers in non-fermented fish products are Gram-negative contaminating bacteria that arrive through poor hygiene. In fermented foods, however, the BA producers belong mainly to the LAB, which form part of the normal microbiota of raw milk (Silla Santos, 1996; Linares et al., 2011). Even adequate hygienic measures do not, therefore, prevent their presence. Moreover, most treatments aimed at reducing the populations of BA producers, such as pasteurization and high pressure treatments, are not fully effective and can have a negative impact on LAB species important in the production of fermented foods (Ladero et al., 2011; Calzada et al., 2013). New methods that specifically target BA-producing bacteria are therefore needed.

Tyramine is one of the BAs most commonly found at undesirably high concentrations in cheese (Fernandez et al., 2007; Linares et al., 2011). In fact, the symptoms (hypertension, headache or even migraine, Ladero et al., 2010a) that may appear after the ingestion of cheese with a high tyramine concentration are together known as the “cheese effect” (ten Brink et al., 1990), and they can be severe in more susceptible people (McCabe-Sellers et al., 2006). Recently, it has been shown that tyramine has a strong cytotoxic effect (Linares et al., 2016) – even stronger than that of histamine, the only BA for which a legal limit has been established in some foods.

The genus *Enterococcus* is responsible for the accumulation of tyramine in some cheese types (Ladero et al., 2010b); *Enterococcus faecalis*, which is prevalent in raw milk, is one of the main culprits (Ladero et al., 2010b; McAuley et al., 2015; O’Sullivan et al., 2015). It is now known that the biosynthesis of tyramine and putrescine (another BA) in this bacterium is a species-level trait (Ladero et al., 2012b).

Bacteriophages (or simply ‘phages’) are viruses that infect and kill bacteria. They are extremely host-specific, commonly infecting just one species and sometimes a reduced number of strains within that species. Their use as a tool against harmful bacteria has therefore been proposed, and certainly, bacteriophage-based biocontrol would appear to have great potential in the improvement of food safety (Garcia et al., 2008; Mahony et al., 2011). Phages also afford other advantages as biocontrol agents: they are harmless to humans, animals and plants, have a long shelf life if stored correctly, are able to resist the environmental stresses encountered during food processing (as well as the physiochemical conditions of food), are relatively cheap and easy to isolate and propagate, and since they are self-replicating and self-limiting, low dosages can be employed since they multiply only, and as long as the host is present (Sillankorva et al., 2012). They have been proposed as a means of eliminating food-borne pathogens such as *Listeria*, *Staphylococci*, and enterotoxigenic *Escherichia coli* strains (Garcia et al., 2008), but they might also be used against food spoilage microorganisms (Deasy et al., 2011; Yamaki et al., 2015). The use of phages that infect BA-producing LAB in fermented foods has, to our knowledge, not been proposed. Bacteriophages that specifically targets BA-producing bacteria could provide a novel and specific way to eliminate these microorganisms while having no impact on those LAB members required for fermentation to proceed. Therefore, we propose to screen, isolate and characterize phages that infect *E. faecalis*, which as mentioned above is the main responsible for the accumulation of tyramine in cheese, with the ultimate goal of using them as biocontrol agents for reducing tyramine in dairy products.

In the present work, the bacteriophage Q69, which infects *E. faecalis*, was isolated from cheese and characterized (host range, morphology, etc.). A proof-of-concept study was then performed to evaluate the use of this phage in reducing BA accumulation in fermented foods. To this general objective, the capacity of Q69 to reduce the production of tyramine in broth was first assayed. Subsequently, its capability to reduce the accumulation of tyramine was tested in an experimental cheese model.

## Materials and Methods

### Materials, Bacterial Strains, and Media

Unless otherwise stated, all reagents were purchased from Sigma–Aldrich (Spain). A cheese made from raw goat milk was purchased from a supermarket and its tyramine content determined. It was then used as a source for phage screening. Different tyramine-producing *E. faecalis* strains (15a, 18a, 19a, 23a, 52c, 63c, V583, and CECT481^T^) were used as potential hosts in phage screening. To determine the host range of the phage Q69, additional *E. faecalis* strains were assayed (**Table 1**). All bacterial strains were grown in M17 broth (Oxoid, Spain) supplemented with 0.5 glucose (GM17) without aeration. In host strain assays, the culture medium was also supplemented with 10 mM CaNO_3_ and 10 mM Mg_2_SO_4_ (CM-GM17). Phage titres were determined in double-layer agar plates, mixing 100 μl of serial dilutions in SM buffer (20 mM Tris-HCl pH 7.5, 1 mM Mg_2_SO_4_, 100 mM NaCl) with 100 μl of an overnight culture of an appropriate host strain. Plates were incubated at 37°C for 18 h and the resulting plaques counted.

**Table 1:** Host range determination for phage Q69.

Origin	*Enterococcus faecalis* strains	Q69 infection	Reference/collection
Type strain	CECT481^T^	+	CECT
Dairy	15a	–	Ladero et al., 2012b
Dairy	18a	+	Ladero et al., 2012b
Dairy	19a	+	Ladero et al., 2012b
Dairy	23a	+	Ladero et al., 2012b
Dairy	28a	–	Ladero et al., 2012b
Dairy	52c	+	Ladero et al., 2012b
Dairy	54c	–	Ladero et al., 2012b
Dairy	57c	+	Ladero et al., 2012b
Dairy	63c	+	Ladero et al., 2012b
Dairy	CECT 4039	–	CECT
Dairy	BA62	–	Ladero et al., 2012b
Dairy	BA64	–	Ladero et al., 2012b
Dairy	V61	–	Ladero et al., 2012b
Dairy	V63	–	Ladero et al., 2012b
Meat	LMG20645	–	LMG
Meat	LMG12161	–	LMG
Human	CECT795	+	CECT
Human	CECT4176	–	CECT
Human	HFS25	–	Ladero et al., 2009
Human	HFS57	+	Ladero et al., 2009
Human	HFS59	+	Ladero et al., 2009
Human	HFS62	–	Ladero et al., 2009
Human	HFS66	–	Ladero et al., 2009
Human	HFS69	–	Ladero et al., 2009
Clinical	JH2-2	–	Giard et al., 1996
Clinical	V583	–	Paulsen et al., 2003

*+ indicates strains sensitive to phage Q69 infection*.

*CECT: Colección Española de Cultivos Tipo*.

*LMG: Laboratorium voor Microbiologie*.

### Tyramine Determination

Tyramine was determined by UHPLC. In broth it was quantified directly, from a 100 μl sample, as described by Ladero et al. (2015). The tyramine in cheese samples was first extracted and quantified as previously described (Herrero-Fresno et al., 2012) with some modifications. Briefly, 1 g of cheese was mixed with 10 ml of 0.1 M HCl containing 0.2% (w/v) 3,3′ thiodipropionic acid using an Ultra Turrax T50 homogeniser (OMNI International, USA) for 2 min at 20,000 rpm. The samples were then disrupted for 30 min in an ultrasonic bath and centrifuged at 5000 × *g* for 30 min. After removing the fat layer, the supernatant was filtered through 0.45 μm PTFE filters (VWR, Spain). The filtrates were deproteinized by precipitation with trichloroacetic acid (12% v/v). They were then kept in ice for 30 min and centrifuged (12,000 × *g* for 10 min) in a 5810 Eppendorf benchtop centrifuge (Eppendorf, Spain), and the supernatant neutralized with NaOH (0.7 N) as described by del Rio et al. (under review). Supernatant samples (100 μL) were then derivatized with diethyl ethoxymethylenemalonate as described by Linares et al. (2013), and the tyramine separated out and quantified in an H-Class Acquity UPLC^TM^ UHPLC system (Waters, USA) running Empower 2 software (Waters), as described by Redruello et al. (2013).

### Isolation of Phages from Cheese Samples

Q69 phages were isolated from the purchased cheese by enrichment culture and by following the standard spot method in double-layer agar plates as follows. One gram of cheese was homogenized for 2 min in 9 ml of 2% sodium citrate in a Lab-Blender 400 stomacher (Seward Ltd., UK). One hundred microlitres of this homogenate were then added to 10 ml of CM-GM17, supplemented with cycloheximide (20 μg/ml) to inhibit yeast and mold growth, and inoculated with 100 μl of an overnight culture of host bacteria (*E. faecalis* strains: 15a, 18a, 19a, 23a, 52c, 63c, V583, or CECT481^T^; **Table 1**). Enrichment cultures were incubated without aeration at 30°C for 24 h. Samples were then placed in tubes and centrifuged (2000 × *g* for 15 min) in an 5810 Eppendorf benchtop centrifuge, and 100 μl of the supernatant obtained added to a new enrichment culture. After two rounds of enrichment, 10 μl were spotted onto double-layered agar CM-GM17 plates and incubated for 24 h at 30°C. When an inhibition halo was observed, the source supernatant was streaked to obtain single plaques. Some of these plaques were individually tested against the targeted host strains. For bacteriophage purification, a single plaque was picked up with a sterile tip, inoculated into 50 ml of MC-GM17 broth inoculated with the host strain, and incubated at 30°C until cell lysis was observed. The culture was then spun (10,000 × *g* for 15 min) in a 7780 centrifuge (Kubota, Korea) with an AG6512C rotor, concentrated using the PEG/NaCl method (Binetti et al., 2005), and stored.

### Electron Microscopy

Concentrated phage particles were further purified in a continuous CsCl gradient by centrifugation (100,000 × *g* for 20 h at 4°C) in an Optimax ultracentrifuge (Beckman Coulter, USA), as described by Gutierrez et al. (2011). Purified phage particles were stained with 2% uranyl acetate solution and electron micrographs produced using a CCD Gatan Erlangshen ES 1000 W camera coupled to a JEOL JEM 1011 transmission electron microscope (JEDL USA Inc, USA) operated at 100 kV (performed at the Electron Microscopy Service of the Biotechnology National Centre [CNB-CSIC], Spain).

### DNA Isolation and Restriction Analysis

Phage DNA was obtained from a concentrated suspension of phage particles following the procedure described by Binetti et al. (2005). Eighty microliters of lysis solution (0.25 M EDTA, pH 8.1; 0.5 M Tris-HCl, pH 9.6; 2.5% sodium dodecyl sulfate) were added to 400 μl of phage suspension and incubated in a water bath at 65°C for 30 min. One hundred microlitres of 8 M potassium acetate were then added, and the mixture incubated on ice for 15 min and further centrifuged (16,000 × *g*, 10 min, at 4°C) in a 5415R Eppendorf centrifuge. Phage DNA was precipitated from the supernatant with one volume of isopropanol, kept at room temperature for 5 min, and centrifuged again (16,000 × *g*, 10 min at room temperature). The pellet was resuspended in TE buffer (10 mM Tris-HCl, 1 mM EDTA, pH 8.0) in the presence of 0.3 M sodium acetate pH 4.8, and precipitated twice with isopropanol for 5 min, followed by centrifugation (16,000 × *g*, 10 min at room temperature). The DNA pellet was washed with absolute ethanol and 70% ethanol before being resuspended in TE buffer.

The purified phage DNA was digested with *Hin*dIII restriction endonuclease (Takara, Japan). Restricted DNA was separated in 0.8% agarose gel in TAE buffer (40 mM Tris-acetate, 1 mM EDTA) and visualized under UV light after ethidium bromide staining using the G-Box system (Syngene, UK).

Microbial DNA from cheese samples was extracted from 1 g of cheese following the method described by Ladero et al. (2012a), which is based on the method of Ogier et al. (2002).

### Real-Time Quantitative PCR

The number of tyramine-producing enterococci in cheese were measured by real-time quantitative PCR (qPCR) using the specific primers tdcE4f and tdcE4r (Ladero et al., 2010b). Reactions were performed using the SYBR Green PCR Master Mix Kit (Applied Biosystems, UK) in 20 μl volumes, which included 1 μl of template, 900 nM of each primer, and 10 μl of SYBR Green PCR Master Mix containing ROX as a passive reference. Amplification and detection were performed using an ABI Prism Fast 7500 sequence detection system (Applied Biosystems) following the standard program. The cycle threshold (Ct) values (automatically assigned by the thermocycler software) from 1/10 dilutions of cheese sample DNA were employed to calculate cell numbers using the equation cited by Ladero et al. (2010b).

### Phylogenetic Analysis

A phylogenetic analysis of the *E. faecalis* bacteriophage sequences available in the NCBI database (**Table 2**) was performed by virtual digestion (with *Hin*dIII endonuclease) and gel separation of their genome sequences, using Vector NTI v9.0 software (Invitrogen, UK). GeneTools software (SynGene) was used to analyze the *Hin*dIII restriction patterns obtained. An UPGMA (unweighted pair-group method with arithmetic mean) dendrogram was produced by profile comparisons based on the MW of generated bands and a position tolerance of 1%.

**Table 2:** Characteristics of the phages subjected to phylogenetic analysis.

Phage	Family	Origin	Lifestyle	Genome size (bp)	Accession number
ECP3	*Myoviridae*	Water	Lytic	145518	KJ801617
EF24C	*Myoviridae*	Water	Lytic	142072	AP009390
EFRM31	*Siphoviridae*	Water	Lytic	16945	GU815339
FL3B	*Siphoviridae*	Clinical	Lysogenic	40275	GQ478087
EF11	*Siphoviridae*	Clinical	Lysogenic	42822	GQ452243
EFACPT1	*Siphoviridae*	Water	Lytic	40923	JX193904
EFAP1	*Siphoviridae*	Farm	Lytic	21115	FJ792813
VD13	*Siphoviridae*	Human	Lytic	55113	KJ094032
IMEEF1	*Siphoviridae*	Water	Lytic	57081	KF192053
BC611	*Siphoviridae*	Clinical	Lytic	53996	AB712291
SAP6	*Siphoviridae*	Water	Lytic	58619	JF731128
EFDG1	*Myoviridae*	Water	Lytic	147589	KP339049
FL1C	*Siphoviridae*	Clinical	Lysogenic	38721	GQ478083
pp1-V583	*Siphoviridae*	Clinical	Lysogenic	37601	AE016830
EF62	*Siphoviridae*	Infant feces	Lysogenic	39505	CP002495
pp3-V583	*Siphoviridae*	Clinical	Lysogenic	48701	AE016830
IMEEF3	*Siphoviridae*	Hospital Water	Lytic	41687	KF728385

### Resistance to Pasteurization

To test the phage capacity to resist a thermal treatment, phage particles (10^6^ pfu ml^-1^) were added to 5 ml of skimmed milk (Oxoid) and subjected to pasteurization (as defined by the IDFA) in a WB-22 water bath (with a microprocessor controller, and integrated auto-diagnostic system, and a temperature resolution of 0.1°C) (Memmert, Germany) at 63°C for 30 min (Ladero et al., 2011). Phage survival was then tested as previously indicated (Madera et al., 2004), using double-layer agar plates and mixing 100 μl of serial dilutions in SM buffer with 100 μl of an overnight culture of *E. faecalis* 23a as the host strain.

### Biocontrol of Tyramine Production: Phage Challenge Assay

CM-GM17 supplemented with 5 mM tyrosine was inoculated with 10^4^ cfu ml^-1^ cells of *E. faecalis* 23a. Phage Q69 was added at different multiplicities of infection (MOI = 0.1, 1 and 10). Inoculated media were incubated at 30°C for 15 h. Viable cells, phage titres and tyramine concentrations were measured and compared with the results for inoculated media in the absence of phages.

### Biocontrol of Tyramine Production in Cheese: Phage Challenge Assay

A tyramine biocontrol trial was also performed in a mini-Cabrales-type cheese. Cheeses were made following the procedure described by del Rio et al. (under review) with the following modifications. Milk was inoculated with 10^4^ cfu ml^-1^ cells of *E. faecalis* 23a and challenged with phage Q69 at an MOI of 0.1. Curd was supplemented with tyrosine (2 mM). Samples were taken from the curd after 60 days of ripening at 16°C. *E. faecalis* cells were then quantified by qPCR, phage particles by serial dilutions spotted onto double-layered agar CM-GM17 plates, and the tyramine produced by UHPLC.

### Statistical Analysis

Means ± standard deviations were calculated from at least three independent results, and compared using the Student’s *t*-test. Significance was set at *p* < 0.05.

## Results

### Bacteriophage Isolation

Phage Q69 was isolated from a cheese made from raw goat milk that contained 249.66 mg kg^-1^ tyramine. After homogenization of the cheese matrix, 100 μl samples were added to CM-GM17 cultures inoculated with one of the eight potential *E. faecalis* host strains. After two rounds of enrichment a growth inhibition halo was observed for the culture supernatants of six *E. faecalis* strains. These supernatants were streaked to obtain isolated plaques. At least three individual plaques from each plate were tested against all the *E. faecalis* strains used in the screening process. All showed the same plaque size and infected the same strains; it was thus assumed that a single phage was isolated. One isolated plaque was obtained after streaking the *E. faecalis* type strain (CECT481^T^) and used to obtain a phage stock for further characterization. The isolated phage was named Q69.

### Bacteriophage Q69 Host Range and Morphology

To determine the host range, 27 *E. faecalis* strains of different origin (**Table 1**) were challenged with phage Q69 via the spot test. The phage was able to infect 10 strains of dairy and human origin (**Table 1**), including the type strain *E. faecalis* CECT 481^T^. In all cases, a clear halo was observed, indicating phage Q69 to be a lytic phage.

**FIGURE 1:**
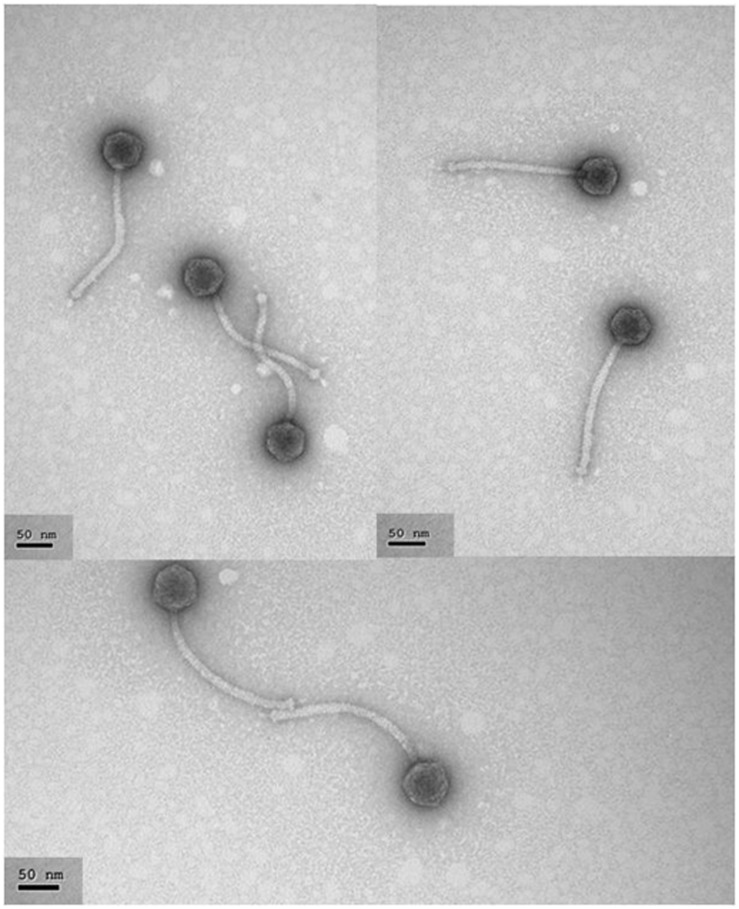
**Electron photomicrographs of phage Q69**. Phage particles were prepared, negatively stained, and examined under the electron microscope as described in Section “Materials and Methods”. The scale bar represents 50 nm.

Photomicrographs of Q69 (**Figure 1**) showed it to belong to the family S*hiphoviridae*; it had a large, non-contractile tail and isometric head. The tail length was estimated at 181 ± 4 nm and the head diameter at 50 ± 1 nm.

### Genetic Characterization of Bacteriopahge Q69

**FIGURE 2:**
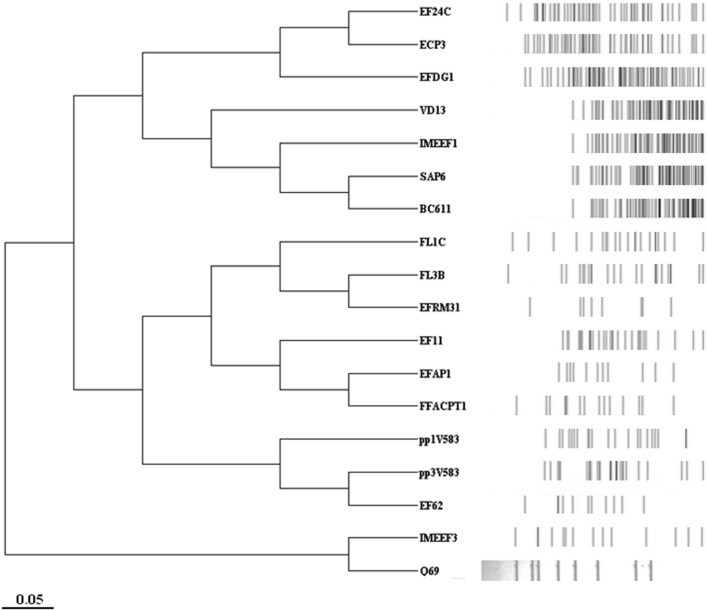
**Phylogenetic tree of *Enterococcus faecalis* bacteriophages**. Phylogenetic analysis of the *Hin*dIII profile of virtually digested *E. faecalis* phage genomes available in databases. The name of each phage is shown (as represented in **Table 2**) as well as its *Hin*dIII restriction profile.

Initial genetic characterization of phage Q69 involved restriction analysis with endonuclease *Hin*dIII. Based on the restriction profile (**Figure 2**), the genome size was estimated at 31,280 bp. Its genome-digested *Hin*dIII profile was compared to other phage genome sequences available in the NCBI database (**Table 2**). The phylogenetic tree revealed no clear association between the *Hin*dIII profiles and functional characteristics such as life style or origin, etc. However, it did indicate phage Q69 to be quite different from most other *E. faecalis* phages with a presence in the NCBI database. The most related phage was IMEEF3, a lytic phage isolated from water supplies/waste water in clinical environments (Li et al., 2014).

### Resistance to Heat Treatment

Since this work contemplated the isolated phage being used as a biocontrol agent in dairy products, its capacity to resist pasteurization was assayed. A phage suspension (10^8^ pfu ml^-1^) in milk was subjected to pasteurization following the indications of the IDFA. The phage titre was not significantly affected by the thermal treatment (63°C for 30 min in a water bath). This suggests that Q69 could resist the same in a dairy environment.

### Phage-Based Tyramine Control in Culture Medium

**FIGURE 3:**
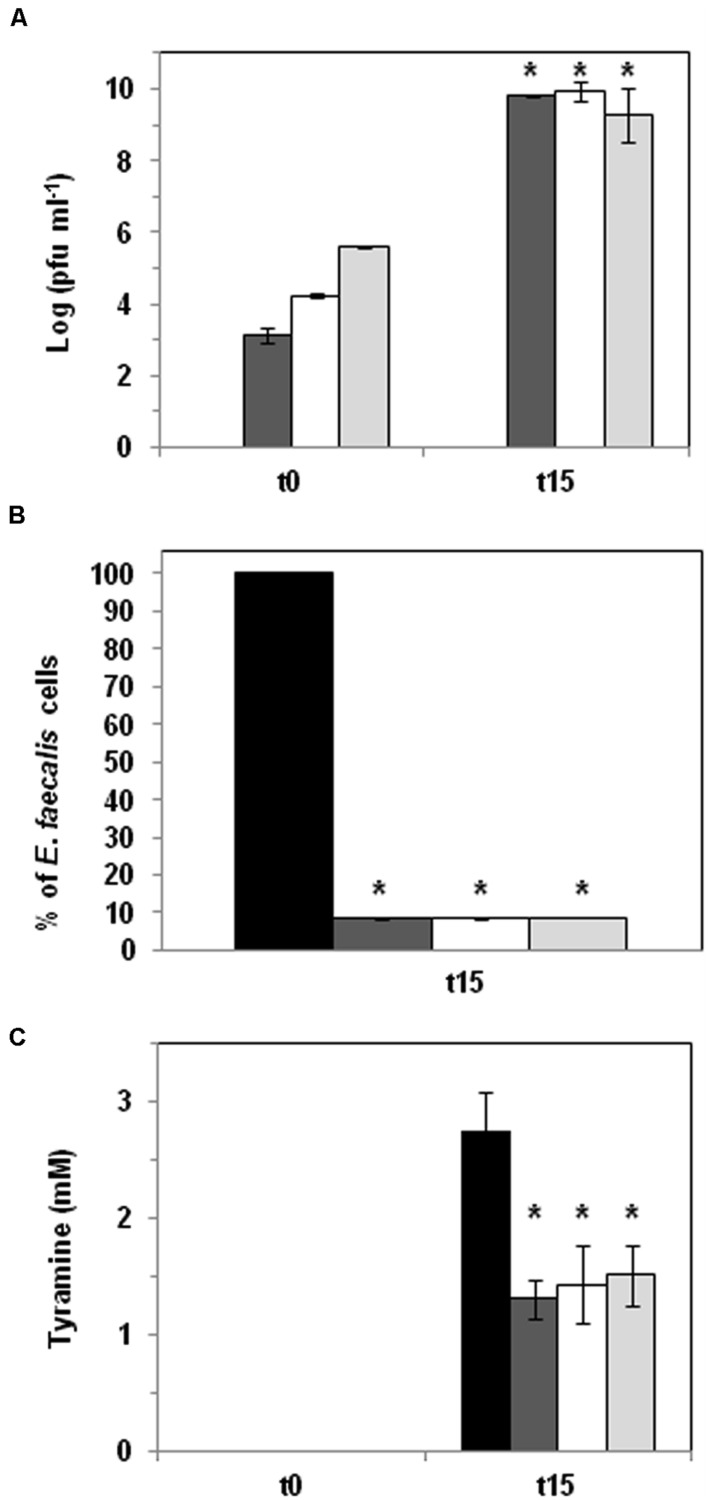
**Phage biocontrol assay in broth medium**. *E. faecalis* was challenged with phage Q69 at three different MOIs (dark gray bar 0.1, white bar 1, and light gray bar 10) in CM-GM17 supplemented with 5 mM tyrosine. Black bars represent control cultures without Q69 phage. **(A)** Number of Q69 phage particles (log pfu ml^-1^) at the beginning of the assay (t0) and after 15 h of incubation (t15). No phages were detected in control cultures (therefore no corresponding black bars appear). An asterisk indicates a significant difference (^∗^*p* < 0.05; Student’s *t*-test) with respect to t0. **(B)** Percentage reduction in *E. faecalis* counts after 15 h of incubation at 30°C. The black bar represents the number of *E. faecalis* 23a cells in control culture without phage Q69 after 15 hours of incubation (100 %). An asterisk indicates a significant difference (^∗^*p* < 0.05; Student’s *t*-test) with respect to control cultures (black bar). **(C)** Tyramine concentration (mM) measured by UPLC at the beginning of the assay (t0) and after 15 hours of incubation (t15). An asterisk indicates a significant difference (^∗^*p* < 0.05; Student’s *t*-test) with respect to control cultures (black bar).

*E. faecalis* 23a was inoculated (10^4^ cfu ml^-1^) into CM-GM17 supplemented with 5 mM tyrosine to ensure substrate availability during the assay. It was then challenged with three different concentrations (MOI = 0.1, 1, and 10) of phage Q69. Cells and phage particles, as well as the tyramine concentration, were measured after 15 h of growth (**Figure 3**). **Figure 3A** shows an increment in the number of phage particles, indicating that phage Q69 was functional and able to replicate under the tested conditions. A significant, concomitant reduction in *E. faecalis* cells was also seen after 15 h at all three MOIs tested (**Figure 3B**). Q69 was in fact able to reduce the proliferation of *E. faecalis* by 90% (counts: 3.05 × 10^9^ cfu ml^-1^ in control culture vs. 4.56 × 10^8^ cfu ml^-1^ in phage treatment culture). The observed reduction in the number of *E. faecalis* cells was reflected in the amount of tyramine produced, since tyramine concentration was a significant 48% lower in the presence of the phage (**Figure 3C**). No differences were seen between phage titres, the number of *E. faecalis* cells, or the amount of tyramine produced at any of the MOIs tested (**Figure 3**), indicating that a low phage concentration (MOI = 0.1) can be used without reducing efficacy.

### Phage-Based Tyramine Control in Cheese

**FIGURE 4:**
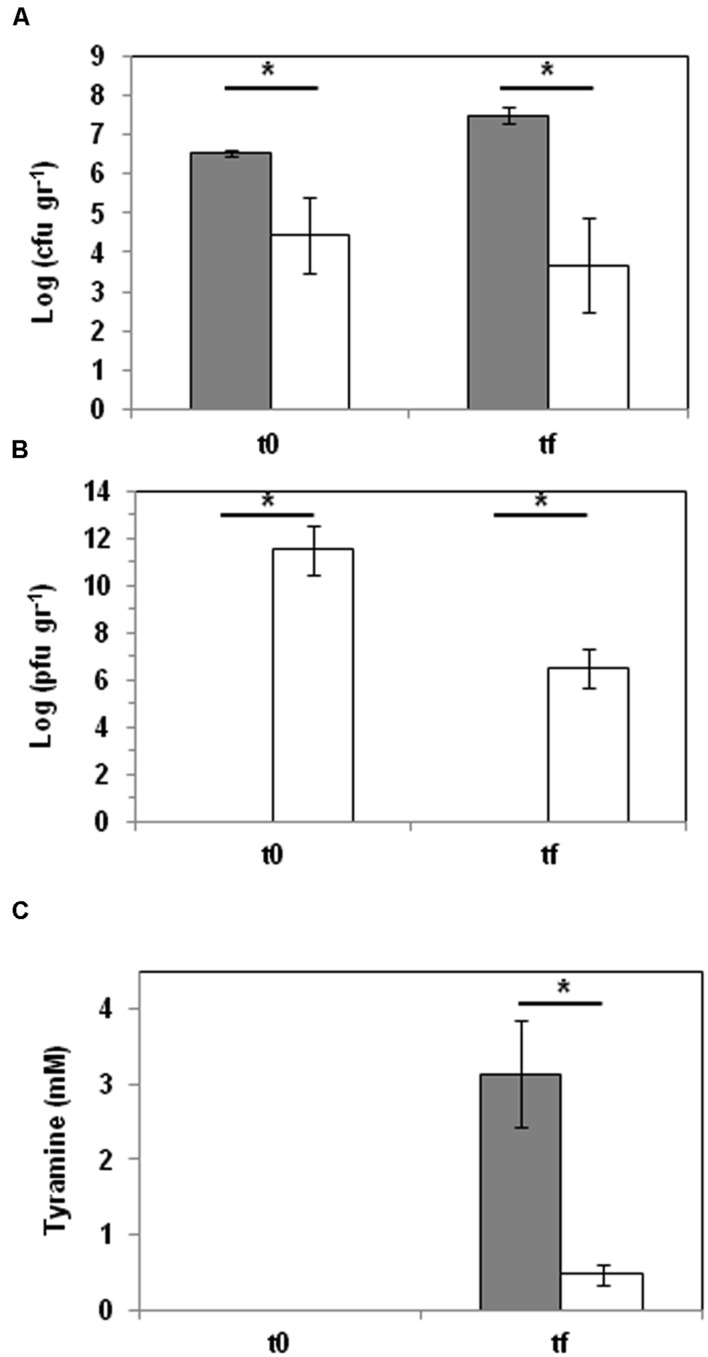
**Phage biocontrol assay in a small-scale cheese model**. *E. faecalis* was challenged with phage Q69 at an MOI of 0.1 (dark gray bar = control cheeses inoculated with *E. faecalis* 23a; white bar = cheeses inoculated with *E. faecalis* 23a and challenged with phage Q69). **(A)** Number of *E. faecalis* cells (log cfu ml^-1^) after manufacturing (t0) and after 60 days of ripening (tf), as calculated by qPCR. **(B)** Number of Q69 phage particles (log pfu ml^-1^) after manufacturing (t0) and after 60 days of ripening (tf). **(C)** Tyramine concentration (mM) measured by UPLC after manufacturing (t0) and after 60 days of ripening (tf). An asterisk indicates a significant difference (^∗^*p* < 0.05; Student’s *t*-test) with respect to control cheeses.

Finally, the capacity of phage Q69 to reduce the accumulation of tyramine was assayed in an experimental cheese model. Milk was inoculated with *E. faecalis* 23a cells (10^4^ cfu ml^-1^) and one batch challenged with phage Q69 at MOI = 0.1 (10^3^ pfu ml^-1^). Samples were taken at the end of manufacture (t0) (i.e., after whey draining and salting), and again at the end of the ripening period (tf) (after 60 days at 12°C) (**Figure 4**). *E. faecalis* cells (determined by qPCR), phage particles (enumerated by serial dilution in double-layer agar plates), and tyramine concentrations (quantified by UPLC) were measured at t0 and tf (**Figure 4**). The presence of phage Q69 significantly reduced the number of tyramine producers, even before ripening (t0, **Figure 4A**), indicating that during milk acidification and curd production it was able to infect and kill *E. faecalis* cells. The increase in phage particles observed at t0 corroborates this hypothesis (**Figure 4B**). The phage particle count was smaller at the end of the ripening period (tf) compared to t0, but a large number (3.7 × 10^6^ pfu g^-1^) was still present and active (**Figure 4B**). At tf, fewer enterococci cells were observed in the cheeses containing the phage (**Figure 4A**). Consequently, tyramine concentration in such cheese was reduced by some 85% (0.46 mM vs. 3.13 mM) with respect to those without Q69 (**Figure 4C**). In summary, the presence of bacteriophage Q69 effectively reduced the accumulation of tyramine in the model cheese.

## Discussion

We have isolated, from a cheese elaborated with raw goat milk that presented a medium tyramine concentration (249.66 mg kg^-1^), a lytic bacteriophage (Q69) infecting *E. faecalis*, one of the main tyramine producers in cheeses elaborated with raw milk (Ladero et al., 2010b; O’Sullivan et al., 2015).

According to its morphology, phage Q69 belongs to the *Siphoviridae* family (**Figure 1**). Most of the LAB-infecting phages isolated so far have belonged to this family, and have been described as a very heterogenic group compared to the members of other families such as *Podoviridae* and *Myoviridae* (Mahony and van Sinderen, 2014; Martínez et al., 2016). Phage Q69 was able to infect 10 of the 27 strains tested; this is within the range observed in other surveys for *E. faecalis* phages of the *Siphoviridae* family (Lee et al., 2014). In general, phages infecting LAB species have a very narrow host range – sometimes just one strain (Kot et al., 2014; Martínez et al., 2016). Phage Q69, however, infected over 35% of the strains tested in this work – strains often of different origin; this might be considered a broad host range for a dairy phage (**Table 1**).

The genome size of phage Q69 was estimated from its *Hin*dIII restriction profile. At 31 Kb it is within the range of other *E. faecalis* phages (**Table 2**). The phylogenetic tree produced using the virtual *Hin*dIII restriction profile of genomes from other *E. faecalis*-infecting phages revealed Q69 to differ from most of them. Similar to findings reported by other authors (Zinno et al., 2010), this restriction profile-based tree allowed no functional groups based on origin, lifestyle or host range, etc. to be recognized. However, it did provide a useful tool for typifying phages based on genome characteristics (Zinno et al., 2010); for example, in the present work the *Myoviridae* phages, which have the largest genomes of all those examined, grouped together. The *Hin*dIII profile of Q69 showed clear differences to most of the other phage genomes examined. Its closest relative appears to be IMEEF3, a phage isolated from hospital sewage (Li et al., 2014). These two phages constitute a group differentiated from all of the rest phages, whose genome sequence is available. However, a more in-depth characterization of Q69 will be needed to compare its genome properly with that of IMEEF3 and other *E. faecalis*-infecting phages, and thus establish a more accurate phylogeny and more fully identify the genetic differences among them.

Phage Q69 was shown a useful tool for reducing tyramine accumulation in both the broth medium- and cheese model-based assays. This effect is clearly mediated by a reduction in the population of tyramine-producing enterococci. In the broth medium it reduced the population of *E. faecalis* 23a by 90% on average, and there was a concomitant fall in tyramine production of some 50% (**Figure 3**). No significant differences were seen between the three phage MOIs assayed (**Figure 3**). The lowest MOI (0.1) was therefore enough to obtain the maximum reduction of *E. faecalis* numbers. The lower the phage concentration that can be used, the lower the costs involved at industrial level.

*E. faecalis* is the predominant enterococcal species in raw milk, in which it can reach concentrations of 10^2^–10^3^ cfu ml^-1^ (McAuley et al., 2015). In the present work, 10^4^ cfu ml^-1^ of tyramine-producing *E. faecalis* were used as the starting concentration, along with a minimum MOI of 0.1 for phage Q69. The finding that this small amount of phage was able to impede the proliferation of *E. faecalis* (**Figure 4**) shows that adding it to raw milk could efficiently reduce *E. faecalis* numbers in cheese products. In fact, the phage added proliferated well, reducing the number of enterococcal cells (**Figure 4**), and the tyramine content (85% lower). Indeed, it reached 3.13 mM in cheeses without Q69 – equivalent to 429.4 mg of tyramine per kg^-1^ of cheese, a value that exceeds the tyramine concentration safety limit (200–500 mg kg^-1^) (Ladero et al., 2010c; Alvarez and Moreno-Arribas, 2014; Linares et al., 2016). In contrast, when the phage was present, the tyramine concentration was held to 63.7 mg kg^-1^, clearly below this limit.

The fact that pasteurization did not significantly reduce the phage Q69 titre would allow its direct addition to raw milk. It could thus start to control the *E. faecalis* population from the very beginning of the manufacturing process, and might even be said to have a complementary effect with pasteurization. It has been suggested that phages would liberate cytosolic enzymes to the food matrix that could continue exerting their activities (Steen et al., 2007; Gardini et al., 2012). Therefore, in the present case, decarboxylating enzymes could continue producing BA. The addition of the phages before pasteurization could avoid this problem by preventing the proliferation of BA producers. Thus, although some decarboxylase activity would be released, it should not be enough to accumulate high BA concentrations. Resistance to pasteurization has been studied in other LAB phages, showing a great variability from sensitive to highly resistant (Binetti and Reinheimer, 2000; Madera et al., 2004; Atamer et al., 2013), but most of them are designed as thermo-resistant phages (Atamer et al., 2013).

At the end of the ripening period, phage particle numbers had fallen somewhat, although they were still high (10^6^ pfu ml^-1^). The presence of active phage particles at this point could help to control the accumulation of tyramine during storage, a critical time during which tyramine-producing enterococci might increase in number if the storage temperature were inadequate (Marcobal et al., 2006; Linares et al., 2012). Foodborne spoilage microorganisms are generally already present in low concentration in food raw materials, and can also arrive as contaminants during its manufacture and storage (Martinez et al., 2011). They can sometimes reach very large numbers, with the negative economic and food quality impacts this entails. If the population of spoilage microorganisms remains controlled at low number their spoilage effect could be maintained under acceptable limits (i.e., the presence of tyramine below the recommended limit of 500 mg Kg^-1^).

The present work provides proof of concept regarding the potential of phages to control spoilage microorganisms that produce BA. It also shows that targeting the tyramine-producing *E. faecalis* with Q69 can reduce tyramine production under different experimental conditions. However, while the results are promising, it should be remembered that more than one strain of *E. faecalis* would probably be present in (or eventually contaminate) any milk sample. The idea that a single phage might be able to infect all the strains present in cheese is therefore too hopeful, especially when dealing with LAB species, the phages of which have a narrow host range (Kot et al., 2014). A cocktail of phages with different host ranges, however, could overcome this problem (Chan et al., 2013). So far, most of the *E. faecalis* phages isolated have targeted host strains of environmental and human origin (**Table 2**); efforts should therefore be made to increase the battery of phages of dairy origin.

In this work, we have isolated from dairy environment, and characterized, the *E. faecalis* bacteriophage Q69. The presence of Q69 efficiently reduced the final concentration of tyramine under different experimental conditions, including an experimental cheese model. In the light of the results obtained, and given that *E. faecalis* is the main microorganism responsible for tyramine accumulation in cheese, we can propose the use of phage Q69, together with other *E. faecalis*-infecting phages, as an effective tool to prevent tyramine accumulation during the manufacture and storage of fermented dairy foods.

## Author Contributions

VL designed, carried out some experiments, and drafted the manuscript; CG-S, ES-L, and BR performed some experiments; BdR, MM, and MF participated in study design and helped to write the manuscript; MA provided the general concept and supervised the work and the manuscript. All authors contributed to the discussion of the research and approved the final manuscript.

## Conflict of Interest Statement

The authors declare that the research was conducted in the absence of any commercial or financial relationships that could be construed as a potential conflict of interest.

## ABBREVIATIONS

BAs: Biogenic amines
CM-GM1: broth supplemented with 0.5 glucose, 10 mM CaNO_3_ and 10 mM Mg_2_SO_4_
GM17: M17 broth supplemented with 0.5 glucose
IDFA: International Dairy Foods Association
LAB: Lactic acid bacteria
UHPLC: ultra-high performance liquid chromatography
